# Treatment of acute mesenteric ischemia between 2010 and 2020 – a German nation-wide study

**DOI:** 10.1186/s12876-023-02926-w

**Published:** 2023-09-06

**Authors:** Stefanie Bette, Osama Habeeballah, Jan H. Luitjens, Thomas Kroencke, Christian Scheurig-Muenkler, Josua A. Decker

**Affiliations:** 1 Diagnostic and Interventional Radiology, Faculty of Medicine, University Hospital Augsburg, University of Augsburg, Stenglinstr. 2, 86156 Augsburg, Germany; 2 Centre for Advanced Analytics and Predictive Sciences (CAAPS), University of Augsburg, Universitätsstr. 2, 86159 Augsburg, Germany

**Keywords:** Acute mesenteric ischemia, SARS-CoV2-pandemic, Nation-wide, Endovascular treatment

## Abstract

**Background:**

Aim of this study was to analyze long-term trends of hospitalizations, treatment regimen and in-hospital mortality of in-patients with acute mesenteric ischemia (AMI) over the past decade and effects of the SARS-CoV2-pandemic.

**Methods:**

We analyzed fully anonymized data from the German Federal Statistical Office of patients with AMI between 2010 and 2020. Besides descriptive analyses of age, gender, in-hospital mortality, comorbidity burden and treatment regimen, multivariable logistic regression analyses were performed to identify independent variables associated with in-hospital mortality and different treatment.

**Results:**

A total of 278,121 hospitalizations (120,667 male [43.4%], mean age 72.1 years) with AMI were included in this study. The total number of hospitalizations increased from 2010 (*n* = 24,172) to 2019 (*n* = 26,684) (relative increase 10.4%). In-hospital mortality decreased over the past decade from 36.6% to 2010 to 31.1% in 2019 (rel. decrease 15.2%). Independent risk factors for in-hospital mortality were older age (OR = 1.03 per year), higher comorbidity burden (OR = 1.06 per point in van Walraven score [vWs]), male gender (OR = 1.07), AMI as a secondary diagnosis (OR = 1.44), and the need for surgical (visceral surgery: OR = 1.38, vascular surgery: OR = 3.33) and endovascular treatment (OR = 1.21). We report a decline in hospitalizations during the first wave of infection in spring 2020 (rel. decrease 9.7%).

**Conclusion:**

In-hospital mortality rate has declined over the past decade, but remains high at above 30%. Older age, increased comorbidity and male gender are independent factors for in-hospital mortality. Hospitalizations requiring vascular surgery are associated with high in-hospital mortality, followed by visceral surgery and endovascular approaches. The first wave of the SARS-CoV2-pandemic in spring 2020 implied a decrease in hospital admissions.

**Supplementary Information:**

The online version contains supplementary material available at 10.1186/s12876-023-02926-w.

## Background

Acute mesenteric ischemia (AMI) is a rare, but life-threatening condition [1]. Previous studies reported a short-term mortality (in-hospital or within 30 days) of about 60% [1]. AMI is defined as an acute discontinuity of blood supply to the bowel which may cause ischemia and intestinal necrosis [2]. Etiology of AMI is either non-occlusive (NOMI) - often diagnosed in critical-ill patients with narrowing of blood vessels or heart failure – or occlusive – due to arterial embolism or thrombosis or venous thrombosis [3]. Early diagnosis and treatment are essential for patient’s outcome [4]. The diagnostic tool of choice is biphasic computed tomography angiography (CTA) with arterial and portal venous phase [5]. Treatment regimen depend on many different factors including for example etiology, disease stage and rate of comorbidities; main goal is to restore blood supply to the bowel, to prevent infections and to resect nonviable portions of the bowel [2, 6]. Besides fluid resuscitation, anticoagulation and administration of antibiotics, there are either surgical or endovascular treatment options [2]. The rate of revascularization procedures for thromboembolic AMI, particularly endovascular treatment, increased in the early 2000s [7] and has been shown to be an appropriate alternative to surgery in selected patients [7–9]. According to recent guidelines, endovascular treatment is considered treatment of choice if available and if there are no complications (e.g. bowel necrosis or infection) that require immediate surgery [2, 10, 11].

In the beginning of 2020, the SARS-CoV-2 pandemic started in Germany; the German ministry of health imposed strict lockdown and hygiene measures to prevent a massive spread of the disease and an overload of the health care system. Previous studies reported – besides the decline of scheduled and elective surgeries and treatments – also a decline of acute medical conditions during the lockdown measures (e.g. for stroke, vascular emergencies, coronary heart disease and emergent general surgery) and an increasing number of complicated diseases and more advanced disease stages [12–22]. A recent study reported constant numbers of visceral surgery emergencies (including mesenteric ischemia and bowel obstruction), including 73 hospitals in Germany [23].

Aims of this study were: (1) to evaluate long-term nationwide trends of treatment regimen, and outcome of acute mesenteric ischemia and the effects of the first year of the SARS-CoV2-pandemic and (2) to identify independent variables associated with in-hospital mortality and different treatment approaches.

## Methods

### Data collection, patient cohort

The data acquisition’s procedure has been reported in detail previously [24]. In brief, the authors wrote syntaxes in Stata 17 (www.stata.com) including all codes according to the International Classification of Diseases, Tenth Revision (ICD-10) and the Operating and Procedure (OPS) and corresponding variables. Codes and variables are described in detail below. Fully anonymized data were kindly provided by the research data center (RDC) of the German Federal Statistical Office (Destatis) [25]. As fully anonymized data were analyzed, no approval was obtained from the Medical Research and Ethics Committee (MREC). Further, no informed consent from patients was necessary. To ensure complete anonymity of data, the RDC censored all variables including < 3 individuals (according to the German data regulations).

According to ICD-10, hospitalizations with the diagnosis of *acute mesenteric ischemia* (K55.0) were recorded. We included all hospitalizations with AMI (K55.0) between 2010 and 2020 as main diagnosis and as secondary diagnosis. Variables including age, gender (male), duration of hospital stay (days) and in-hospital mortality were recorded for all hospitalizations as well as for subgroups defined according to treatment regimens.

We categorized the following treatment regimens according to specific OPS-codes: endovascular treatment, vascular surgery and visceral surgery. OPS-codes are listed in Supplemental Table 1. Conservative treatment was defined as the absence of all listed OPS-codes.

We further assessed the burden of comorbidities by analyzing all secondary diagnoses of the Elixhauser groups and also calculating the Elixhauser score (sum of positive Elixhauser groups) [26, 27] and the weighted linear van Walraven score (vWs) as described previously [28].

In a separate analysis, we analyzed the time frame between hospital admission and treatment for AMI for both – all hospitalizations and AMI as main diagnosis as well as for subgroups with different treatment regimen.

To assess the impact of the pandemic, we examined and compared semimonthly (01.-14. and 15.-end of month) data for all hospitalizations due to AMI in the years 2019 and 2020.

### Statistical analyses

Statistical analyses were performed using SPSS 28.0 (IBM statistics), syntaxes were written in Stata 17 (www.stata.com). We present data either as absolute numbers (n) and percentages (%), as mean (± standard deviation [sd]) or as median (interquartile range [IQR]), as indicated. We calculated absolute and relative changes between 2010 and 2019 and between 2019 and 2020. Univariable and multivariable logistic regression analyses were performed including age, gender, vWS, secondary diagnosis and type of treatment as parameters and in-hospital mortality as outcome. Logistic regression analyses with different treatment regimen as outcome were performed including the parameters age, gender, secondary diagnosis, vWS and in-hospital mortality. Odds ratios are presented with 95% confidence intervals. *P*-values < 0.05 were considered to indicate statistically significance.

## Results

A total of 278,121 hospitalizations (120,667 male [43.4%], mean age 72.1 years) with *acute mesenteric ischemia* were analyzed between 2010 and 2020. AMI as main diagnosis was reported in 134,843 (48.5%) of hospitalizations.

### Trends between 2010 and 2019

**Fig. 1:**
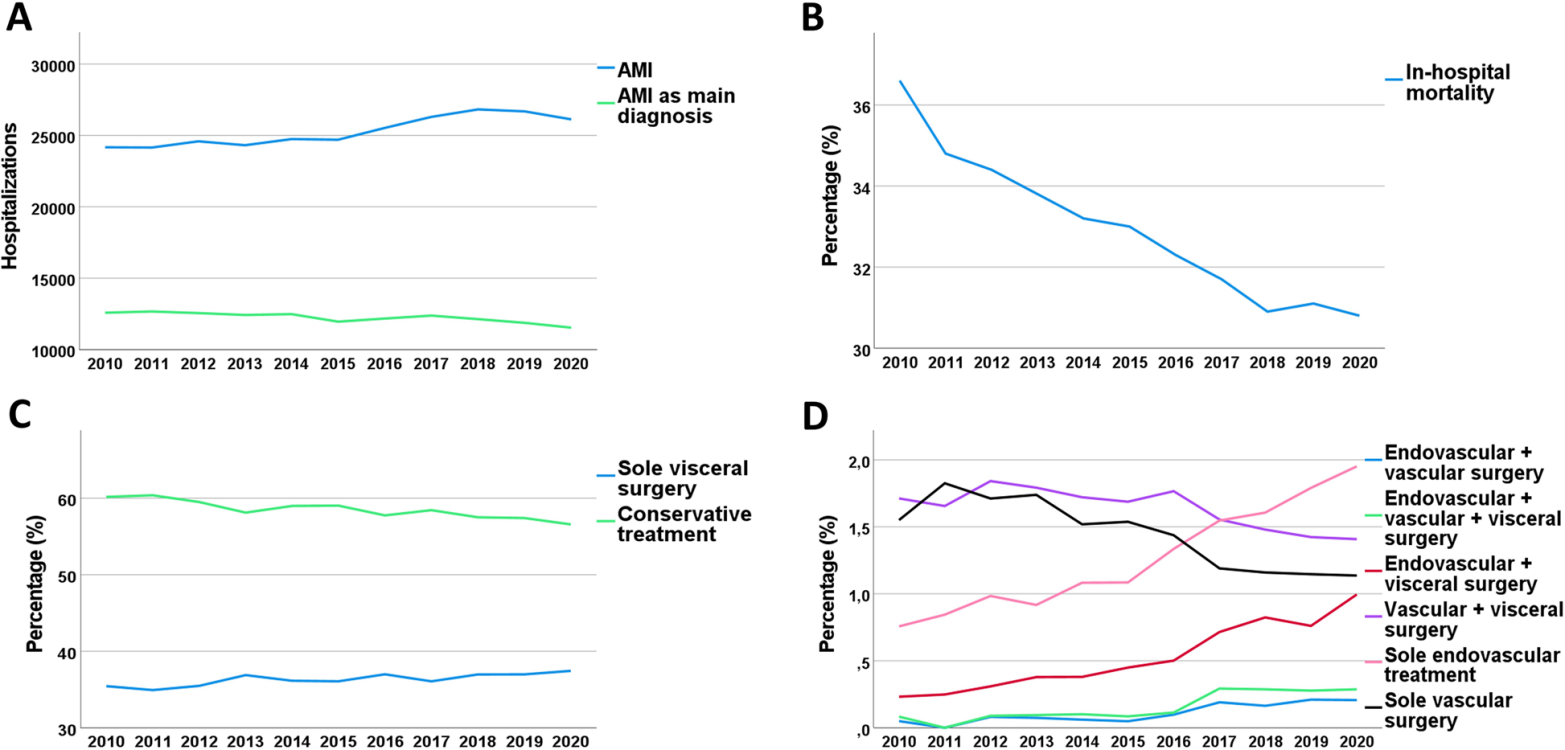
Trends in admissions for acute mesenteric ischemia (AMI) in Germany between 2010 and 2020. **A** Long-term trend of hospitalizations for AMI as main and secondary diagnosis **B** in-hospital mortality, **C** + **D** trends for different treatment regimen

Between 2010 and 2019, 251,997 hospitalizations (108,712 male [43.1], mean age 72.2 years) were analyzed. Detailed descriptive patient characteristics including in-hospital mortality, length of in-hospital stay, comorbidity burden and treatment regimen are shown in Table 1; Fig. 1.

**Table 1:** Hospitalizations due to AMI –patient characteristics between 2010 and 2019 according to treatment regimen and in-hospital mortality

**All hospitalizations, n**	251,997			
Age, mean (± sd)	72.2 ± 15.1			
Men, n (%)	108,712 (43.1)			
In-hospital death, n (%)	83,453 (33.1)			
In-hospital stay, d, median (IQR)	11 (5-21)			
vWS, median (IQR)	10 (5-17)			
vWS, mean (±sd)	11.1 ± 9.4			
Elixhauser score, median (IQR)	3 (2-5)			
Elixhauser score, mean (±sd)	3.6 ± 2.3			
	**Endovascular**	**Vascular surgery**	**Visceral surgery**	**Conservative treatment**
**All hospitalizations, n**	4,922	8,544	97,042	147,926
Age, mean (± sd)	70.1 ± 12.4	71.9 ± 13.2	70.6 ± 15.7	73.2 ± 14.8
Men, n (%)	2,510 (51.0)	3,889 (45.5)	46,550 (48.0)	58,945 (39.8)
In-hospital death, n (%)	1,772 (36.0)	5,012 (58.7)	35,111 (36.2)	45,292 (30.6)
In-hospital stay, d, median (IQR)	14 (6-27)	15 (5-30)	18 (10-33)	7 (3-14)
vWS, median (IQR)	12 (6-19)	13 (8-20)	12 (5-19)	8 (2-16)
vWS, mean (±sd)	13.3 ± 9.6	14.6 ± 9.1	13.0 ± 9.7	9.8 ± 9.0
Elixhauser score, median (IQR)	4 (3-6)	4 (3-6)	4 (2-6)	3 (2-5)
Elixhauser score, mean (±sd)	4.4 ± 2.3	4.5 ± 2.1	4.1 ± 2.3	3.3 ± 2.2
**AMI as main diagnosis, n**	2,765	5,047	33,732	85,422
Age, mean (± sd)	71.4 ± 11.9	74.6 ± 12.5	73.3 ± 13.5	74.9 ± 14.0
Men, n (%)	1,301 (47.1)	2,076 (41.1)	15.162 (44.9)	28,156 (33.0)
In-hospital death, n (%)	793 (28.7)	2,805 (55.6)	11.798 (35.0)	21,093 (24.7)
In-hospital stay, d, median (IQR)	11 (4-22)	14 (3-26)	15 (8-26)	6 (2-10)
vWS, median (IQR)	10 (5-17)	12 (7-18)	11 (5-17)	5 (0-12)
vWS, mean (±sd)	11.3 ± 8.6	13.0 ± 8.3	11.9 ± 8.7	7.4 ± 7.5
Elixhauser score, median (IQR)	4 (2-5)	4 (3-5)	4 (2-5)	3 (1-4)
Elixhauser score, mean (±sd)	4.0 ± 2.2	4.2 ± 2.1	3.9 ± 2.2	2.8 ± 2.0
**In-hospital death**				
	**Yes**		**No**	
**All hospitalizations, n**	83,453		168,544	
Age, mean (± sd)	76.2 ± 12.8		70.2 ± 15.8	
Men, n (%)	36,763 (44.1)		71,949 (42.7)	
In-hospital stay, d, median (IQR)	6 (2-17)		12 (7-23)	
vWS, median (IQR)	14 (8-21)		8 (2-15)	
vWS, mean (±sd)	14.9 ± 9.6		9.2 ± 8.7	
Elixhauser score, median (IQR)	4 (3-6)		3 (2-5)	
Elixhauser score, mean (±sd)	4.3 ± 2.3		3.3 ± 2.2	
**AMI as main diagnosis, n**	34,561		88,739	
Age, mean (± sd)	80.1 ± 10.4		72.3 ± 14.3	
Men, n (%)	12,392 (35.9)		32,641 (36.8)	
In-hospital stay, d, median (IQR)	2 (1-7)		9 (5-15)	
vWS, median (IQR)	11 (5-17)		5 (0-12)	
vWS, mean (±sd)	11.8 ± 8.5		7.6 ± 7.7	
Elixhauser score, median (IQR)	3 (2-5)		3 (1-4)	
Elixhauser score, mean (±sd)	3.5 ± 2.2		3.0 ± 2.1	

We observed a 10.4% increase in hospitalizations between 2010 (*n* = 24,172) and 2019 (*n* = 26,684). The rate of endovascular treatment increased until 2019, both for patients receiving only endovascular treatment and for those who received combined endovascular and surgical treatment. Sole endovascular treatment increased from *n* = 183 (0.8%) in 2010 up to *n* = 478 (1.8%) in 2019 (relative increase 136.6%). The rate of conservative management decreased from 60.2% in 2010 to 57.4% in 2019 (relative decrease 4.6%). Also, the rate of sole vascular surgery decreased until 2019 (1.1%) from 1.6% to 2010 (relative decrease 26.1%). Both, the rate of in-hospital mortality and duration of in-hospital stay decreased over time: in-hospital mortality relatively declined by 15.2% from 36.6% to 2010 to 31.1% in 2019 and in-hospital stay by 9.1% from a median of 11 days to a median of 10 days. Comorbidity burden increased over time by 14.3% (e.g. mean Elixhauser score 3.3 in 2010 to 3.8 in 2019) (Table 2).

In univariable analyses, all parameters were significantly associated with outcome and therefore included in multivariable analyses. Using in-hospital mortality as outcome, age (Odds Ratio [OR] 1.03 [95% confidence interval 1.03–1.03], *P* < 0.001), male gender (OR 1.07 [1.06–1.09], *P* < 0.001), vWS (OR 1.06 [1.06–1.06], *P* < 0.001), AMI as secondary diagnosis (OR 1.44 [1.41–1.47], *P* < 0.001) and treatment regimen (endovascular: OR 1.21 [1.12–1.31], *P* = 0.002, visceral surgery: OR 1.38 [1.28–1.48], *P* < 0.001, vascular surgery: OR 3.33 [3.14–3.52], *P* < 0.001) were identified as significant prognostic factors (Table 3). For different treatment regimen as outcome, the following parameters were highlighted: AMI as secondary diagnosis as parameter for visceral surgery as outcome showed an OR of 2.17 (95% CI 2.13–2.21, *P* < 0.001), whereas OR was below 1.00 for endovascular treatment (OR 0.57 [0.54–0.61], *P* < 0.001) and for vascular surgery (OR 0.44 [0.42–0.46], *P* < 0.001). Results of all parameters are shown in Table 3.

**Table 2:** Hospitalizations due to AMI – comparison between 2010, 2019 and 2020

Year	2010	2019	2020	Absolute change 2010 - 2019	Relative change 2010 - 2019	Absolute change 2019 - 2020	Relative change 2019 - 2020
**All hospitalizations, n**	24,172	26,684	26,124	+2,512	**+10.4%**	-560	**-2.1%**
Age, mean (± sd)	72.93 (±14.9)	71.47 (±15.4)	71.20 (±15.4)	-1	**-2.0%**	0	**-0.4%**
Men, n (%)	10,053 (41.6)	12,014 (45.0)	11,955 (45.8)	+1,961 (+3.4)	**+8.3%**	-59 (+0.7)	**+1.6%**
In-hospital death, n (%)	8,852 (36.6)	8,287 (31.1)	8,050 (30.8)	-565 (-5.6)	**-15.2%**	-237 (-0.2)	**-0.8%**
In-hospital stay, d, median (IQR)	11 (5-22)	10 (5-21)	10 (4-20)	-1	**-9.1%**	0	**0.0%**
**Treatment:**							
endovascular + vascular surgery	12 (0.0)	56 (0.2)	54 (0.2)	+44 (+0.2)	**+322.7%**	-2 (0.0)	**-1.5%**
endovascular + visceral and vascular surgery	20 (0.1)	74 (0.3)	75 (0.3)	+54 (+0.2)	**+235.2%**	1 (0.0)	**+3.5%**
endovascular + visceral surgery	56 (0.2)	203 (0.8)	260 (1.0)	+147 (+0.5)	**+228.4%**	57 (+0.0)	**+30.8%**
vascular + visceral surgery	414 (1.7)	380 (1.4)	368 (1.4)	-34 (-0.3)	**-16.9%**	-12 (0.0)	**-1.1%**
conservative treatment	14,546 (60.2)	15,320 (57.4)	14,781 (56.6)	+774 (-2.8)	**-4.6%**	-539 (-0.8)	**-1.5%**
endovascular	183 (0.8)	478 (1.8)	510 (2.0)	+295 (+1.0)	**+136.6%**	32 (+0.2)	**+9.0%**
vascular surgery	375 (1.6)	306 (1.1)	297 (1.1)	-69 (-0.4)	**-26.1%**	-9 (0.0)	**-0.9%**
visceral surgery	8,566 (35.4)	9,867 (37.0)	9,779 (37.4)	+1,301 (+1.5)	**+4.3%**	-88 (+0.5)	**+1.2%**
**Elixhauser domains, n (%):**							
Hypertension (uncomplicated)	11,188 (46.3)	13,962 (52.3)	13,740 (52.6)	+2,774 (+6.0)	**+13.0%**	-222 (+0.3)	**+0.5%**
Hypertension (complicated)	1,959 (8.1)	2,047 (7.7)	1,965 (7.5)	+88 (-0.4)	**-5.3%**	-82 (-0.1)	**-1.9%**
Kidney disease	5,658 (23.4)	6,474 (24.3)	6,290 (24.1)	+816 (+0.9)	**+3.7%**	-184 (-0.2)	**-0.8%**
Cardiac arrhythmias	7,739 (32.0)	8,462 (31.7)	8,409 (32.2)	+723 (-0.3)	**-1.0%**	-53 (+0.5)	**+1.5%**
Diabetes (complicated)	3,942 (16.3)	4,468 (16.7)	4,341 (16.6)	+526 (+0.4)	**+2.7%**	-127 (-0.1)	**-0.8%**
Diabetes (uncomplicated)	2,052 (8.5)	1,910 (7.2)	1,906 (7.3)	-142 (-1.3)	**-15.7%**	-4 (+0.1)	**+1.9%**
Congestive heart failure	5,675 (23.5)	6,054 (22.7)	5,881 (22.5)	+379 (-0.8)	**-3.4%**	-173 (-0.2)	**-0.8%**
Fluid and electrolyte disorders	11,222 (46.4)	14,837 (55.6)	15,207 (58.2)	+3,615 (+9.2)	**+19.8%**	370 (+2.6)	**+4.7%**
Chronic pulmonary disease	2,885 (11.9)	3,400 (12.7)	3,336 (12.8)	+515 (+0.8)	**+6.8%**	-64 (0.0)	**+0.2%**
Hypothyroidism	1,916 (7.9)	3,890 (14.6)	4,110 (15.7)	+1,974 (+6.7)	**+83.9%**	220 (+1.2)	**+7.9%**
Obesity	1,547 (6.4)	1,837 (6.9)	1,881 (7.2)	+290 (+0.5)	**+7.6%**	44 (+0.3)	**+4.6%**
Valvular disease	1,821 (7.5)	2,226 (8.3)	2,351 (9.0)	+405 (+0.8)	**+10.7%**	125 (+0.7)	**+7.9%**
Depression	1,297 (5.4)	1,751 (6.6)	1,707 (6.5)	+454 (+1.2)	**+22.3%**	-44 (0.0)	**-0.4%**
Coagulopathy	5,014 (20.7)	6,290 (23.6)	6,297 (24.1)	+1,276 (+2.8)	**+13.6%**	7 (+0.5)	**+2.3%**
Other neurological disorders	733 (3.0)	666 (2.5)	666 (2.5)	-67 (-0.5)	**-17.7%**	0 (+0.1)	**+2.1%**
Paralysis	1,237 (5.1)	1,223 (4.6)	1,213 (4.6)	-14 (-0.5)	**-10.4%**	-10 (+0.1)	**+1.3%**
Weight loss	936 (3.9)	2,940 (11.0)	2,748 (10.5)	+2,004 (+7.1)	**+184.5%**	-192 (-0.5)	**-4.5%**
Deficiency anemia	544 (2.3)	1,048 (3.9)	1,011 (3.9)	+504 (+1.7)	**+74.5%**	-37 (-0.1)	**-1.5%**
Rheumatoid arthritis/collagen disorders	424 (1.8)	585 (2.2)	563 (2.2)	+161 (+0.4)	**+25.0%**	-22 (0.0)	**-1.7%**
Alcohol abuse	763 (3.2)	987 (3.7)	1,028 (3.9)	+224 (+0.5)	**+17.2%**	41 (+0.2)	**+6.4%**
Pulmonary circulation disorders	819 (3.4)	1,169 (4.4)	1,199 (4.6)	+350 (+1.0)	**+29.3%**	30 (+0.2)	**+4.8%**
Liver disease	1,964 (8.1)	3,622 (13.6)	3,549 (13.6)	+1,658 (+5.4)	**+67.1%**	-73 (0.0)	**+0.1%**
Solid tumor without metastasis	2,362 (9.8)	3,176 (11.9)	2,988 (11.4)	+814 (+2.1)	**+21.8%**	-188 (-0.5)	**-3.9%**
Blood loss anemia	770 (3.2)	1,145 (4.3)	1,082 (4.1)	+375 (+1.1)	**+34.7%**	-63 (-0.1)	**-3.5%**
Metastatic cancer	853 (3.5)	1,438 (5.4)	1,415 (5.4)	+585 (+1.9)	**+52.7%**	-23 (0.0)	**+0.5%**
Psychoses	179 (0.7)	207 (0.8)	181 (0.7)	+28 (0.0)	**+4.8%**	-26 (-0.1)	**-10.7%**
Drug abuse	377 (1.6)	144 (0.5)	183 (0.7)	-233 (-1.0)	**-65.4%**	39 (+0.2)	**+29.8%**
Lymphoma	140 (0.6)	169 (0.6)	164 (0.6)	+29 (+0.1)	**+9.4%**	-5 (0.0)	**-0.9%**
Peptic ulcer disease, excluding bleeding	197 (0.8)	161 (0.6)	155 (0.6)	-36 (-0.2)	**-26.0%**	-6 (0.0)	**-1.7%**
AIDS / HIV	6 (0.0)	10 (0.0)	14 (0.1)	+4 (0.0)	**+51.0%**	4 (0.0)	**+43.0%**
**Elixhauser score, median (IQR)**	3 (2-5)	4 (2-5)	4 (2-5)	+1	**+33.3%**	0	**0.0%**
**Elixhauser score, mean (±sd)**	3.3 ± 2.2	3.8 ± 2.4	3.9 ± 2.4	+0.5	**+14.3%**	0.0	**+1.3%**
**vWS, median (IQR)**	9 (4-16)	10 (5-18)	10 (5-18)	+1	**+11.1%**	0	**0.0%**
**vWS, mean (±sd)**	10.1 ± 8.6	11.9 ± 9.9	12.0 ± 9.8	+1.8	**+18.4%**	+0.1	**+0.8%**

A subgroup analysis of patients after excluding cases in which a life-threatening AMI is less likely (patients that were discharged alive after conservative treatment) was performed. Considering this fact, in-hospital mortality was 55.9% for all hospitalizations between 2010 and 2019 (Supplemental Table 2). Regarding annual data, we reported a decrease of in-hospital mortality from 59.6% in 2010 to 53.0% in 2019 (rel. change – 11.2%) (Supplemental Table 3).

#### Effect of the SARS-CoV2-pandemic

During 2020, a 2.1% decrease of hospitalizations was observed (*n* = 26,124 in 2020 vs. *n* = 26,684 in 2019). The rate of conservative management further decreased (56.6% in 2020 vs. 57.4% in 2019, relative decrease of 1.5%), whereas the rate of endovascular treatment further increased (2.0% in 2020 vs. 1.8% in 2019). While comorbidity burden was slightly higher in 2020 compared to 2019 (mean Elixhauser score 3.9 vs. 3.8, relative increase 1.3%), the rate of in-hospital mortality further decreased in 2020 (30.8% in 2020 vs. 31.1% in 2019, relative decrease 0.8%).

In 2019, 23.4% of patients requiring endovascular procedures, were treated immediately, 16.4% within 12 h and 9.2% within 24 h resulting in a total of 48.9% receiving treatment within the first 24 h after hospital admission. In 2020 in contrast, 26.8% received treatment immediately, 15.6% within 12 h, 14.2% within 24 h resulting in a total of 56.6% treatment within the first 24 h.

**Fig. 2:**
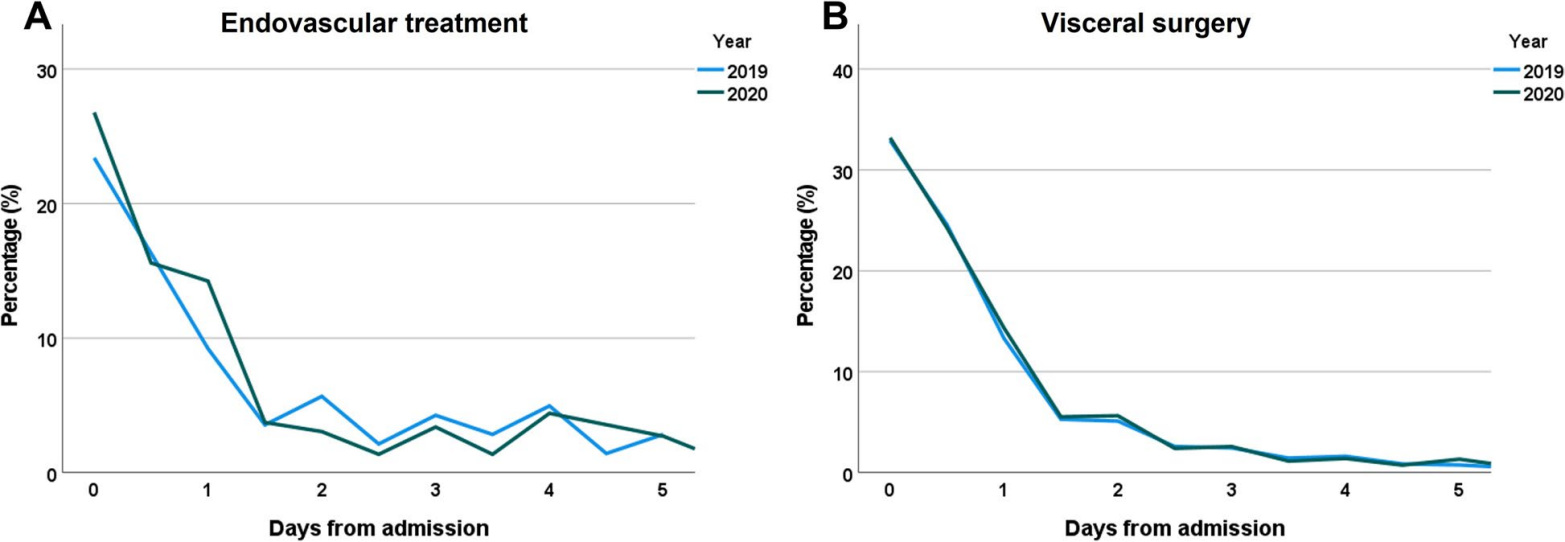
Time frame between hospital admission and treatment in patients with acute mesenteric ischemia as main diagnosis in 2019 and 2020

For patients requiring visceral surgery during 2019, 33.1% were treated immediately and a total of 70.9% within 24 h. In 2020, similar data were observed: treatment of 33.2% immediately and a total of 71.1% within 24 h after hospital admission (Fig. 2).

**Fig. 3:**
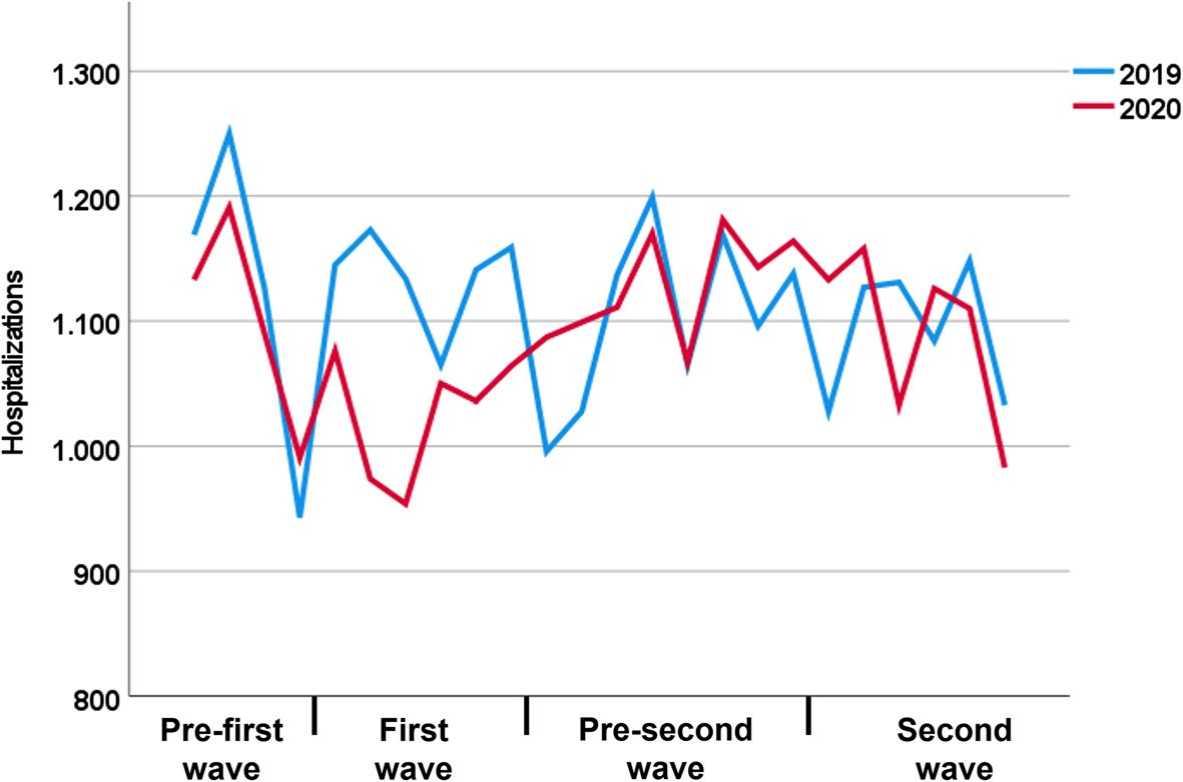
Hospitalizations for acute mesenteric ischemia in 2019 compared to the waves of infection of the first year of the SARS-CoV2-pandemic in 2020

Especially during the first wave of infection in spring 2020 we saw a dramatic decrease in hospitalizations for AMI (-9.1% compared to 2019) (Table 4; Fig. 3). During the second wave of infection, there was no further decrease in hospitalizations and no rebound effect of admissions missing during the first wave was observed.

**Table 3:** Multivariable logistic regression analyses for acute mesenteric ischemia

**In-hospital death as outcome**			
	**Odd’s ratio**	**95% CI**	***P*** **-Value**
Main diagnosis (ref)	1.00		
Secondary diagnosis	1.44	1.41-1.47	<0.001
Age (continuously)	1.03	1.03-1.03	<0.001
Gender			
Female (ref)	1.00		
Male	1.07	1.06-1.09	<0.001
vWS (continuously)	1.06	1.06-1.06	<0.001
Type of treatment			
Conservative treatment (ref)	1.00		
Endovascular treatment	1.21	1.12-1.31	0.002
Visceral surgery	1.38	1.28-1.48	<0.001
Vascular surgery	3.33	3.14-3.52	<0.001
**Treatment as outcome**			
Main diagnosis (ref)	1.00		
Secondary diagnosis	1.98	1.95-2.01	<0.001
Age (continuously)	0.99	0.99-0.99	<0.001
Gender			
Female (ref)	1.00		
Male	1.15	1.13-1.17	<0.001
vWS (continuously)	1.03	1.03-1.03	<0.001
Survival (ref)	1.00		
In-hospital death	1.11	1.09-1.13	<0.001
**Visceral surgery as outcome**			
Main diagnosis (ref)	1.00		
Secondary diagnosis	2.17	2.13-2.21	<0.001
Age (continuously)	0.99	0.99-0.99	<0.001
Gender			
Female (ref)	1.00		
Male	1.14	1.12-1.16	<0.001
vWS (continuously)	1.03	1.03-1.03	<0.001
Survival (ref)	1.00		
In-hospital death	1.05	1.03-1.07	<0.001
**Vascular surgery as outcome**			
Main diagnosis (ref)	1.00		
Secondary diagnosis	0.44	0.42-0.46	<0.001
Age (continuously)	0.99	0.99-0.99	<0.001
Gender			
Female (ref)	1.00		
Male	1.04	0.99-1.08	0.121
vWS (continuously)	1.04	1.03-1.04	<0.001
Survival (ref)	1.00		
In-hospital death	2.95	2.81-3.09	<0.001
**Endovascular treatment as outcome**			
Main diagnosis (ref)	1.00		
Secondary diagnosis	0.57	0.54-0.61	<0.001
Age (continuously)	0.99	0.99-0.99	<0.001
Gender			
Female (ref)	1.00		
Male	1.29	1.22-1.37	<0.001
vWS (continuously)	1.03	1.03-1.03	<0.001
Survival (ref)	1.00		
In-hospital death	1.09	1.02-1.16	<0.001

**Table 4:** Hospitalizations due to AMI in 2019 and 2020 in correlation to the two waves of the SARS-CoV2-pandemic in 2020

	2019	2020	Absolute change	Rel. change
**Hospitalizations**				
Pre-first wave (Jan-Feb)	4,488	4,405	**-83**	**-1.8%**
First wave (March-May)	6,817	6,154	**-663**	**-9.7%**
Pre-second wave (June-Sep)	8,828	9,022	**+194**	**+2.2%**
Second wave (Oct-Dec)	6,551	6,543	**-8**	**-0.1%**

## Discussion

The present study reports nation-wide data of 278,121 hospitalizations with acute mesenteric ischemia between 2010 and 2020. The major findings are: (1) Between 2010 and 2019, an increase in hospitalizations for AMI was observed; while the still low rate of endovascular treatment (about 1–2%) increased steadily over the past decade, the remarkably high rate of conservative treatment (about 60%) and the low rate of vascular surgery (about 1–2%), as well as in-hospital mortality, decreased. (2) Older age, male gender, higher comorbidity burden, AMI as secondary diagnosis, and treatment (especially vascular surgery) were independently associated with increased in-hospital mortality. (3) A decrease in hospitalizations due to AMI was observed during the first wave of the SARS-CoV2-pandemic.

The trends that we observed between 2010 and 2019 are consistent with previously reported data from the United States [29]: increasing number of hospitalizations for AMI, increasing rate of endovascular treatment, and decreasing in-hospital mortality. The decline in in-hospital mortality might be explained by the continuous improvement of treatment regimen, early diagnosis, and risk factor reduction [29]. However, despite the decline of in-hospital mortality during the past decades, we still observed a very high rate of in-hospital mortality of about 30%. Also, the shown trends with an increasing rate of endovascular treatment procedures might overstate the reported results. With a rate of about 2%, endovascular treatment is carried out in a very small proportion of patients with acute mesenterial ischemia.

Restoring blood flow to the bowel is critical [2, 30]. Endovascular treatment of AMI has grown in popularity over the past decades [2, 31, 32]. However, the decision between surgical and endovascular treatment is controversial; mainly due to the lack of randomized controlled trials, the different pathophysiological mechanisms of the disease and the resulting heterogeneous patient cohorts [2, 33–37]. According to current guidelines, endovascular treatment is the preferred treatment option when available and when there is no suspicion of complications (e.g. bowel necrosis or peritonitis) [2, 8, 11]. Decision for primary endovascular treatment requires the availability of this procedure and the absence of bowel necrosis or infection as it might otherwise delay surgical treatment or mediate further inflammatory processes due to bowel reperfusion. As clinical symptoms of mesenteric ischemia are often unspecific, many patients are diagnosed in an advanced disease stage. These considerations might explain the still low rate of endovascular treatment. The results of our study show a very small portion of patients receiving endovascular treatment (about 1–2%) with slightly increasing numbers over the past decade and a small portion of patients receiving surgical revascularization with a slight decrease over time. These findings might indicate an undertreatment of patients with AMI; however, our data lack clinical data (including disease stage, severity of symptoms, etiology of AMI) and we can therefore not evaluate the cause of the small number of patients receiving revascularization.

Previous studies described lower mortality rates in patients receiving endovascular treatment compared to open surgery [2, 11]. However, there might be a selection bias: patients with more advanced disease stages (including bowel ischemia and peritonitis) more likely receive open surgery and do also have higher morbidity and mortality rates [2].

In our study, most patients with invasive treatment received visceral surgery (about 35–37%). Visceral surgery might be preferentially performed in patients with complications (e.g. bowel ischemia, perforation or peritonitis), disease stages in which revascularization cannot save a necrotic bowel segment. However, an additional surgical or endovascular revascularization may save other segments which are ischemic, but without established necrosis, even after resection of the necrotic segments.

Our data showed a remarkably high rate of conservative treatment (about 60%) with a slight decrease over time. From this portion of patients treated conservatively, about 70% were discharged alive. These findings might point out the uncertainty of the diagnosis in this subgroup and suggest the presence of a large portion of patients with acute on chronic ischemia with different disease severity, either with mild symptom deterioration of an underlying chronic disease, or with more severe complications prompting visceral surgery, or even very advanced disease stages where the patient is considered inoperable for revascularization or bowel surgery and palliation is the only remaining option. To account for this potential bias, we performed a subgroup analysis and excluded all patients that were discharged alive after conservative treatment. This led to a significant aggravation of mortality in the resulting AMI cohort.

Main independent risk factors for in-hospital mortality were higher age, a higher comorbidity burden as well as the need of endovascular or surgical treatment. Previous studies also described age and comorbidity as risk factors for mortality and morbidity [38, 39]. The need for intervention is usually associated with more advanced stages of disease and may therefore explain the higher in-hospital mortality.

This study also included patients with a secondary diagnosis of AMI. AMI can occur in a variety of medical conditions, primarily due to other abdominal diseases (e.g., small bowel obstruction, infection). This is also reflected in our multivariable logistic regression analysis, that shows a high association between AMI as secondary diagnosis and decision for visceral surgery (OR 2.17). Controversially, patients with AMI as secondary diagnosis have a higher probability to receive visceral surgery instead of endovascular treatment and vascular surgery.

Similar to other studies that reported a decrease in hospitalizations for emergent general surgery and other emergent conditions during the pandemic, we also observed a decline for acute mesenteric ischemia [15, 17, 18, 21, 40–43]. In the present study, this decrease was only present during the first wave of infection in spring 2020, whereas no effect was shown during the second wave of infection in late autumn / winter 2020. Similar results were also reported in a nation-wide study from Germany that analyzed hospitalizations for stroke [12]. These findings were attributed to a reduced fear of infection, improved hygiene measures in hospitals and encouragement of patients to seek medical help also in times of the pandemic [12, 44]. Several other studies reported delays of treatment during the SARS-CoV2-pandemic resulting in partially more advanced and more severe disease stages [14, 21, 23]. These delays in treatment might be associated with poorer outcomes. Interestingly, we did not observe an increase in in-hospital mortality during 2020 compared to 2019. The present study reports no effects on treatment timing for patients with visceral surgery and only slight, but opposite effects for patients requiring endovascular procedures. Compared to 2019, a higher proportion of patients received endovascular treatment during the first hours after hospital admission. A potential explanation might be the reallocation of hospital resources with reduction of capacities for surgical treatment resulting in a higher proportion of patients receiving endovascular procedures.

More pronounced effects were observed on numbers of hospitalizations which might explain the missing effect on in-hospital mortality. It might be assumed that a part of patients with symptoms of acute mesenteric ischemia did not seek medical care due to fear of infection during hospitalization. However, our study does not include data on outpatient treatment of patients with mild symptoms and data on prehospital mortality of patients with fulminant disease. As described above, a significant proportion of patients with AMI are not diagnosed alive, which may also bias the reported in-hospital mortality results [45].

This study has limitations: (1) The data that are reported in this study do not include clinical parameters (e.g. disease stage, bowel ischemia, peritonitis, sepsis) and the etiology of AMI (e.g. arterial embolism, venous thrombosis). Therefore, the study cohort may be heterogeneous, which introduces an unavoidable bias especially in the logistic regression analyses. Patients with more advanced disease stages require surgery and are at higher risk of in-hospital mortality. Further (prospective) studies including also clinical data and analyzing subgroups of patients with arterial / venous or non-occlusive AMI are necessary to gather valuable data and to address this bias. (2) Healthcare data are manually collected and assessed to obtain financial remuneration; we cannot exclude coding errors and bias due to economical reasons. (3) This study assesses hospitalizations and not individual patient data. Therefore, there might be inclusion of patients that were admitted two or more times during the observed time period for the same diagnosis. In contrast to chronic medical conditions (e.g. diabetes mellitus) that require repeated hospital admissions, acute mesenteric ischemia is an emergent medical condition and therefore we can assume that only a small cohort of patients might be included more than once. However, there might be a considerable portion of patients with acute on chronic mesenteric ischemia (reflected by the high rate of conservative treatment with relatively low mortality) that require several readmissions. The German classification system differentiates between both entities (ICD-10 code K55.0 for acute mesenterial ischemia and K55.1 for chronic mesenterial ischemia). Our analysis only included hospitalizations with the code K55.0; however, cases with acute on chronic ischemia might be classified by the code K55.0 in most cases, which might introduce an unavoidable bias. Intentional misclassification is unlikely because both clinical codes in combination with identical procedure codes trigger the same reimbursement.

## Conclusions

Our nation-wide study including patients with AMI over the past decade reports increasing numbers of hospitalizations and a decrease in in-hospital mortality, which, however, remains at a high level of over 30%. With AMI as the primary diagnosis, older age, increased comorbidity, and male gender have been shown to be independent factors for in-hospital mortality. Hospitalizations in which vascular surgery is performed are associated with high in-hospital mortality, followed by visceral surgery and endovascular approaches. Endovascular treatment, either alone or in combination with visceral surgery, increased, however, it is still reserved for only a small number of patients, which may be due to late diagnosis and lack of availability in many cases. The first year of the SARS-CoV2-pandemic resulted in a decrease of hospitalizations, however not in increased in-hospital mortality and a delay of treatment.

### Supplementary Information

**Additional file 1: Supplemental Table 1.** Specific OPS-codes for endovascular and surgical (visceral, vascular) treatment regimens.

**Additional file 2: Supplemental Table 2.** Subgroup analysis after excluding patients in whom a life-threatining AMI is less likely.

**Additional file 3: Supplemental Table 3.** Subgroup analysis after excluding patients in whom a life-threatening AMI is less likely.

## Data Availability

The datasets used and/or analyzed during the current study are available from the corresponding author on reasonable request.

## Abbreviations

AMI: Acute mesenteric ischemia
CTA: Computed tomography angiography
ICD-10: International Classification of Diseases, Tenth Revision
IQR: Interquartile Range
MREC: Medical Research and Ethics Committee
OPS: Operating and Procedure System
OR: Odds Ratio
RDC: Research data center
SD: Standard deviation
vWS: Van Walraven score
