# Supplementary material for: Treatment of acute mesenteric ischemia between 2010 and 2020 – a German nation-wide study
Source: BMC Gastroenterol. 2023 Sep 6;23:300. doi: 10.1186/s12876-023-02926-w (PMC10481516; doi:10.1186/s12876-023-02926-w)
Supplement: Supplementary file 1 — Additional file 1: Supplemental Table 1. Specific OPS-codes for endovascular and surgical (visceral, vascular) treatment regimens. [file 12876_2023_2926_MOESM1_ESM.docx]

| **Supplemental Table 1 Specific OPS-codes for endovascular and surgical (visceral, vascular) treatment regimens** | |
| --- | --- |
| **Treatment** | **OPS-code** |
| *Vascular Surgery* | - 5-380.60 - 5-380.61 - 5-380.63 - 5-380.65 - 5-380.66 - 5-380.9g - 5-380.9h - 5-381.60 - 5-381.61 - 5-381.63 - 5-381.65 - 5-381.66 - 5-382.65 - 5-382.60 - 5-382.61 - 5-382.63 - 5-382.66 - 5-382.9g - 5-382.9h - 5-383.60 - 5-383.61 - 5-383.63 - 5-383.65 - 5-383.66 - 5-383.9g - 5-383.9h - 5-388.60 - 5-388.61 - 5-388.63 - 5-388.65 - 5-388.66 - 5-388.9g - 5-388.9h - 5-395.60 - 5-395.61 - 5-395.63 - 5-395.65 - 5-395.66 - 5-395.9g - 5-395.9h - 5-396.6 - 5-396.66 - 5-396.60 - 5-396.61 - 5-396.63 - 5-393.39 - 5-393.49 |
| *Visceral surgery* | - 5-454 - 5-455 - 5-456 |
| *Endovascular treatment* | - 8-836.0a - 8-836.1a - 8-836.2a - 8-836.3a - 8-836.6a - 8-836.7a - 8-836.8a - 8-836.pa - 8-840.0a - 8-840.1a - 8-840.2a - 8-840.3a - 8-840.4a - 8-840.5a - 8-841.0a - 8-841.1a - 8-841.2a - 8-841.3a - 8-841.4a - 8-841.5a - 8-842.0a - 8-842.1a - 8-842.2a - 8-842.3a - 8-842.4a - 8-842.5a - 8-843.0a - 8-843.1a - 8-843.2a - 8-843.3a - 8-843.4a - 8-843.5a - 8-844.0a - 8-844.1a - 8-844.2a - 8-844.3a - 8-844.4a - 8-844.5a - 8-845.0a - 8-845.1a - 8-846.0a - 8-846.1a - 8-848.0a - 8-848.1a - 8-848.2a - 8-848.3a - 8-848.4a - 8-848.5a - 8-84b.0a - 8-84b.1a - 8-84b.2a - 8-84b.3a - 8-84b.4a - 8-84b.5a - 8-836.fa - 8-836.ga - 8-836.ha - 8-836.ja |
