# Supplementary material for: Treatment of acute mesenteric ischemia between 2010 and 2020 – a German nation-wide study
Source: BMC Gastroenterol. 2023 Sep 6;23:300. doi: 10.1186/s12876-023-02926-w (PMC10481516; doi:10.1186/s12876-023-02926-w)
Supplement: Supplementary file 2 — Additional file 2: Supplemental Table 2. Subgroup analysis after excluding patients in whom a life-threatining AMI is less likely. [file 12876_2023_2926_MOESM2_ESM.docx]

| **Supplemental Table 2 Subgroup analysis after excluding patients in whom a life-threatining AMI is less likely** | |
| --- | --- |
| **Hospitalizations between 2010 and 2019** | |
| All patients, n (%)  Discharged alive, n (%)  In-hospital death, n (%)  Endovascular treatment, n (%)  Vascular surgery, n (%)  Visceral surgery, n (%)  Conservative treatment and death in hospital, n (%) | 149,363 (100.0)  65,910 (44.1)  83,453 (55.9)  4,922 (3.3)  8,544 (5.7)  97,042 (65.0)  45,292 (30.3) |
