# Supplementary material for: Treatment of acute mesenteric ischemia between 2010 and 2020 – a German nation-wide study
Source: BMC Gastroenterol. 2023 Sep 6;23:300. doi: 10.1186/s12876-023-02926-w (PMC10481516; doi:10.1186/s12876-023-02926-w)
Supplement: Supplementary file 3 — Additional file 3: Supplemental Table 3. Subgroup analysis after excluding patients in whom a life-threatening AMI is less likely. [file 12876_2023_2926_MOESM3_ESM.docx]

| **Supplemental Table 3 Subgroup analysis after excluding patients in whom a life-threatening AMI is less likely** | | | | | |  |  |
| --- | --- | --- | --- | --- | --- | --- | --- |
| **Year** | **2010** | **2019** | **2020** | **Absolute change**  **2010 - 2019** | **Relative change**  **2010 - 2019** | **Absolute change**  **2019 - 2020** | **Relative change**  **2019 - 2020** |
| **All hospitalizations, n**  All patients, n  Discharged alive, n (%)  In-hospital death, n (%)  **Treatment**  -endovascular + vascular surgery, n (%)  -endovascular + visceral and vascular surgery, n (%)  -endovascular + visceral surgery, n (%)  -vascular + visceral surgery, n (%)  -endovascular, n (%)  -vascular surgery, n (%)  -visceral surgery, n (%)  -conservative treatment and death in hospital, n (%) | 14,844  5,992 (40.4)  8,852 (59.6)  12 (0.1)  20 (0.1)  56 (0.4)  414 (2.8)  183 (1.2)  375 (2.5)  8,566 (57.7)  5,218 (35.2) | 15,642  7,355 (47.0)  8,287 (53.0)  56 (0.4)  74 (0.5)  203 (1.3)  380 (2.4)  478 (3.1)  306 (2.0)  9,867 (63.1)  4,278 (27.3) | 15,444  7,394 (47.9)  8,050 (52.1)  54 (0.3)  75 (0.5)  260 (1.7)  368 (2.4)  510 (3.3)  297 (1.9)  9,779 (63.3)  4,101(26.6) | +798  +1,363 (+6.7)  -565 (-6.7)  +44 (+0.3)  +54 (+0.3)  +147 (+0.9)  -34 (-0.4)  +295 (+1.8)  -69 (-0.6)  +1,301 (+5.4)  -940 (-7.8) | **+5.4%**  **+16.5%**  **-11.2%**  **+342.9%**  **+251.1%**  **+244.0%**  **-12.9%**  **+147.9%**  **-22.6%**  **+9.3%**  **-22.2%** | -198  +39 (+0.9)  -237 (-0.9)    -2 (0.0)  +1 (0.0)  +57 (+0.4)  -12 (0.0)  +32 (+0.2)  -9 (0.0)  -88 (+0.2)  -177 (-0.8) | **-1.3%**  **+1.8%**  **-1.6%**  **-2.3%**  **+2.7%**  **+29.7%**  **-1.9%**  **+8.1%**  **-1.7%**  **+0.4%**  **-2.9%** |
